# Two modified colonoscopically guided fecal microbiota transplantation catheter placement methods: a retrospective study (with video)

**DOI:** 10.3389/fmed.2025.1641325

**Published:** 2025-10-03

**Authors:** Xinxu Li, Qinghua Liu, Yajing Wang, Hongrui Zhang, Zhi Wu, Rui Fan, Yuyi Ma, Wei Gong, Xiaxi Li

**Affiliations:** ^1^Department of Gastroenterology, Shenzhen Hospital, Southern Medical University, Shenzhen, China; ^2^The First School of Clinical Medicine, Nanfang Hospital, Southern Medical University, Guangzhou, China; ^3^Shenzhen Clinical Medical College, Southern Medical University, Shenzhen, China; ^4^The Second School of Clinical Medicine, Zhujiang Hospital, Southern Medical University, Guangzhou, China

**Keywords:** fecal microbiota transplantation, retrospective study, colonoscopes, catheter, methods

## Abstract

**Introduction:**

Fecal microbiota transplantation (FMT) transfers fecal microbiota from a healthy person into a patient for the treatment of various diseases. This study introduces two modified colonoscopically guided fecal microbiota transplantation catheter placement methods and evaluates their effectiveness and safety in clinical use.

**Methods:**

This study retrospectively reviewed medical records and corresponding endoscopist operational records of FMT patients at Shenzhen Hospital, Southern Medical University, from January 13, 2022, to July 26, 2024. The study analyzed 117 cases, divided into the Direct Loop Clamping (DLC) group and the Clip Loop Binding (CLB) group. The primary outcome was the catheter placement success rate. The secondary outcomes were operation-related times and adverse events.

**Results:**

Both groups achieved a 100% success rate in catheter placement. The two methods showed no significant differences in cecal intubation time, withdrawal time, and total operation time. What’s more, the CLB group had a slightly shorter time for the first endoscopic clip securement (median 1.8 min vs. 3.7 min, *P* = 0.006). There were no significant differences in the incidence of adverse events between the two groups, and no severe adverse events were reported.

**Conclusion:**

Both modified colonoscopically guided fecal microbiota transplantation catheter placement methods demonstrated safety and effectiveness in securing the FMT catheter, meeting the needs of patients requiring multiple FMT treatments over a short period. However, further validation through large-scale randomized controlled trials is needed.

## 1 Introduction

Fecal microbiota transplantation (FMT) is a novel treatment that transfers fecal microbiota from healthy donors into recipients (1). FMT has demonstrated efficacy in the clinical treatment of at least 85 different diseases (2), showing good therapeutic effects in treating acute Clostridium difficile infections (3) and chronic inflammatory bowel diseases (4) [such as ulcerative colitis (5) and Crohn’s disease (6)]. The potential applications of FMT are not limited to the gastrointestinal tract; it has also shown promising results in the treatment of extra-intestinal dysbiosis-related diseases (7) [such as autism (8), non-alcoholic fatty liver disease (9), and cancer (10)].

Fecal microbiota transplantation can be delivered through three main routes: the upper, middle, and lower gastrointestinal tracts (11). In clinical practice, the lower gastrointestinal route has been associated with higher patient acceptance and satisfaction (12). Currently, the colonic transendoscopic enteral tubing (TET) technique has been reported as an effective and safe method (13). This technique involves securing a catheter at the ileocecal region with endoscopic clips during colonoscopy to facilitate the subsequent injection of fecal microbiota suspension (14).

Compared with the transendoscopic enteral tubing (TET) technique, which requires two colonoscope insertions and relies on dedicated, relatively expensive TET catheters, this study introduces two modified methods that are equally effective and safe, while also offering material cost-efficiency and ease of adoption by a broader range of endoscopists. In both methods, a single-step catheter placement under colonoscopic guidance directly targets the ileocecal region, utilizing standard gastric tubes. Before inserting the catheter into the colon, we have two methods to secure the catheter to the colonoscope. The first method, which involves directly clamping the catheter’s loop with a single-use hemostatic clip, is termed the “Direct Loop Clamping” (DLC) method. The second method, which involves pre-tying the catheter’s loop to the single-use hemostatic clip, is termed the “Clip Loop Binding” (CLB) method. The principles of these two catheter preparation methods are like those of an improved endoscopically guided nasojejunal tube preparation (15). This study assesses the effectiveness and safety of these two methods for FMT catheter placement in clinical practice, aiming to provide practical alternative options for FMT treatment.

## 2 Patients and methods

### 2.1 Subjects

This single-center, retrospective study was approved by the Medical Ethics Committee of Shenzhen Hospital, Southern Medical University (No. NYSZYYEC20210037) and was conducted in accordance with the Helsinki Declaration. We retrospectively reviewed the medical records and corresponding endoscopist operational records of patients who underwent fecal microbiota transplantation at the Endoscopy Center, Department of Gastroenterology, Shenzhen Hospital, Southern Medical University, from January 13, 2022, to July 26, 2024.

### 2.2 Inclusion and exclusion criteria

#### 2.2.1 Inclusion criteria for patients

1. Age ≥ 3 years.
2. Tolerance to colonoscopy and requirement for FMT.

#### 2.2.2 Exclusion criteria for patients

1. Severe colonic diseases, such as stenosis, fistulas, or perforation risk.
2. Complex perianal or ileocecal junction conditions that may impair colonoscopy.
3. Colonic wall conditions are unsuitable for endoscopic clip securement.
4. Inability to undergo bowel preparation.

#### 2.2.3 Inclusion criteria for endoscopists

1. Proficiency in performing colonoscopies over 3 years, with more than 1000 colonoscopies previously conducted.
2. Have experience with one or both of our modified colonoscopically guided fecal microbiota transplantation catheter placement methods.

#### 2.2.4 Exclusion criteria for endoscopists

1. The operations of colonoscopically guided FMT catheter placement are less than 5.

In this study, the medical records and corresponding endoscopist operational records of the patients must meet both the patient and endoscopist inclusion criteria for final statistical analysis.

### 2.3 Operation of modified colonoscopically guided catheter placement methods

Our modified methods placed a catheter (Ruben 12# with guide wire, 0.035′′ × 1172 mm, Guangzhou Ruben Biotechnology Co., Ltd., Guangzhou, China) in the colon for fecal microbiota transplantation. Standard preoperative preparations included fasting for 4–6 h, abstaining from water for 2 h, and bowel preparation with polyethylene glycol electrolyte solution (PEG), sodium potassium magnesium sulfate, or sodium picosulfate. Patients could independently choose whether to undergo anesthesia. The FMT catheter was placed by an experienced endoscopist with the assistance of a nurse. Before cecal intubation, the catheter was secured to the colonoscope for subsequent operations. In accordance with previous studies, we used two large endoscopic clips to secure the FMT catheter (16).

**FIGURE 1:**
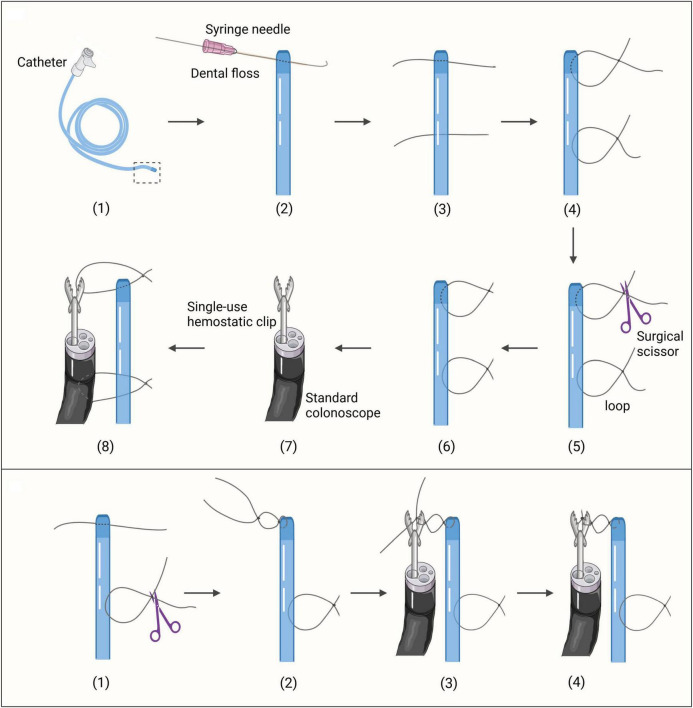
The preparation of the securement methods. **(Top)** The preparation of the DLC securement method. **(Bottom)** The preparation of the CLB securement method. The initial procedures follow steps (1)-(3) as depicted in the top section of Figure 1.

For the Direct Loop Clamping (DLC) group, the preparation involved puncturing the tip of a catheter and threading a dental floss to create surgical knots, followed by trimming the excess to form a loop approximately 2 cm in diameter. A second puncture site was made 5 cm away to create a similar loop. A single-use hemostatic clip [Anrei AMH-HCG-230-135, 2.8 mm minimum applicable channel and 230 cm length, Anrei Medical Devices (Hangzhou) Co., Ltd., Hangzhou, China] was inserted through the colonoscope channel to grasp the first loop of the catheter (Figure 1; Supplementary Video 1).

For the Clip Loop Binding (CLB) group, after puncturing the catheter tip, a dental floss approximately 10 cm in length was left. A knot was tied without cutting the excess, forming a loop with a diameter of 0.5 cm. A second puncture site was made 5 cm away, and a loop of 2 cm in diameter was created. A single-use hemostatic clip was inserted through the colonoscope channel. With the clip open, the first loop was positioned against one side of it, and surgical knots were tied with the remaining dental floss to secure it in place. The loop and clip were then bound together before cecal intubation (Figure 1; Supplementary Video 2).

**FIGURE 2:**
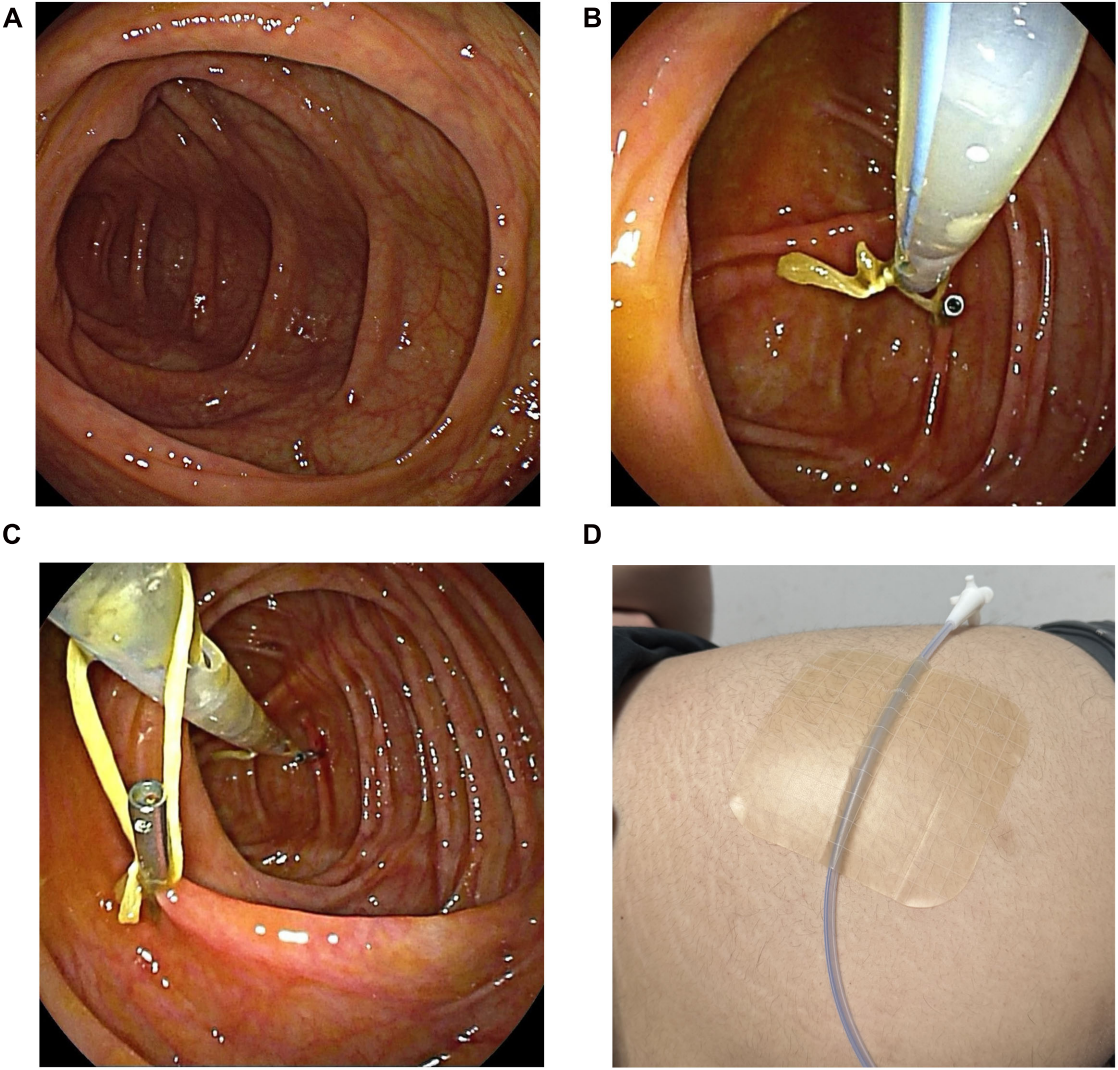
The procedure of the colonoscopically guided catheter placement method. **(A)** Under colonoscopic guidance, the FMT catheter reaches the ileocecal region. **(B)** Secure the first loop to the colonic wall. **(C)** Secure the second loop to the colonic wall. **(D)** Secure the distal end of the FMT catheter to the skin of the buttock.

After the preparation of the catheter was completed, the colonoscope was lubricated, and cecal intubation was performed, with both the colonoscope and the FMT catheter reaching the ileocecal region simultaneously. In both the DLC and CLB groups, once the FMT catheter reached the ileocecal region, an examination during colonoscope withdrawal was conducted (Figure 2A). Once the colonic condition was confirmed to be good, the first loop of the FMT catheter was secured to the colonic wall using an endoscopic clip (i.e., a single-use hemostatic clip) (Figure 2B). Subsequently, the colonoscope was moved to the second loop, and the single-use hemostatic clip was used to grasp and secure this loop to the colonic wall (Figure 2C). After these procedures, the nurse needed to stabilize the FMT catheter to prevent displacement due to friction during colonoscope withdrawal. The endoscopist then slowly withdrew the colonoscope. Finally, the distal end of the FMT catheter was secured to the buttock skin with medical tape (Figure 2D), and the fecal microbiota suspension injection could be performed.

The fecal microbiota transplantation treatment course in our center lasts for 24–72 h. The fecal microbiota suspension for transplantation is prepared by mixing collected feces (≥50 g) with sterile saline at a ratio of 500 ml per 100 g of feces in the GenFMTer. The mixture is centrifuged at 700 *g* for 3 min, and the supernatant is discarded. This centrifugation and washing process is repeated three times. The final suspension, with a volume ratio of 1:2 precipitate to vector solution, is ready for use or storage at −80 °C with 10% glycerol and contains a viable bacterial count of greater than 5 × 10^10^/mL (17). For patients weighing less than 40 kg, two doses of fecal microbiota suspension are administered via the catheter: the first immediately after placement (Day 0) and the second on Day 1. For patients weighing more than 40 kg, four doses are administered: the first immediately after placement (Day 0), the second on Day 1, the third on Day 2, and the fourth on Day 3, before catheter removal. Each dose consists of 50 mL of freshly prepared suspension, derived from donor stool and used within 6 h to preserve microbial viability.

### 2.4 Data collection and outcome measurement

Basic clinical characteristics were collected, including age, sex, body mass index, disease, and whether anesthesia was used. The primary outcome of this study was the catheter placement success rate, defined as the success rate of securing the FMT catheter at the ileocecal region after the colonoscope carrying the catheter reached the ileocecal region. Secondary outcomes included operation-related times: (1) Cecal intubation time, which was the time taken for the colonoscope carrying the FMT catheter to reach the ileocecal region through the anus; (2) First endoscopic clip securement completion time, which was the time required to secure the first endoscopic clip on the colonic wall; (3) Withdrawal time, which was the time taken for the colonoscope to withdraw from the ileocecal region to the anus after the first endoscopic clip was secured, including the operation of securing the second endoscopic clip; (4) Total operation time, which was the duration from the colonoscope’s insertion at the anus until the completion of the FMT catheter placement operation and the colonoscope’s removal from the anus. Additionally, adverse events (AEs) such as catheter dislodgement, diarrhea, perianal pain, abdominal pain, fever, hematochezia, and abdominal distension were monitored. This study evaluated the effectiveness and safety of the colonoscopically guided FMT catheter placement methods. Effectiveness was measured by the catheter placement success rate. Safety was assessed by monitoring the occurrence of adverse events.

### 2.5 Statistical analysis

Data analysis was performed using IBM SPSS Statistics software, version 27 (IBM Corp., Armonk, NY, United States). Prior to data analysis, the normality of variable distributions was assessed. For quantitative variables with a distribution that met the criteria for normality, the independent samples *t*-test was utilized, and results were reported as mean ± standard deviation. For quantitative variables with a distribution that did not meet the criteria for normality, the Mann-Whitney U non-parametric test was applied to assess differences between the two groups, with results presented as median [interquartile range (IQR)]. Categorical variables were compared using the chi-square test and Fisher’s exact test, with results reported as percentages. A *P*-value of less than 0.05 (two-tailed) was considered to indicate statistical significance.

## 3 Results

### 3.1 Basic clinical characteristics

**FIGURE 3:**
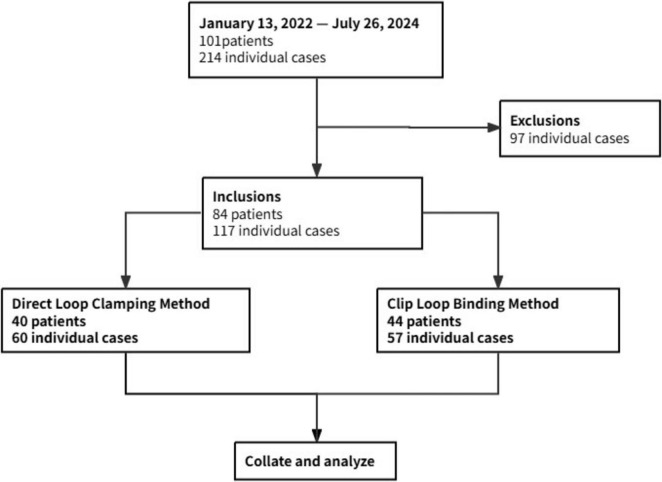
Flowchart of the grouping.

As shown in Figure 3, a total of 101 patients underwent fecal microbiota transplantation (FMT) via our modified colonoscopically guided FMT catheter placement methods, involving 214 individual cases (including multiple FMT treatments in the same patient). After excluding 97 cases due to incomplete data records by the endoscopists during the catheter placement procedure, 117 eligible cases involving 84 patients were included in the analysis. Prior to providing FMT treatment services to patients, our endoscopy center conducted a 40-min training session for endoscopists, introducing the two modified colonoscopically guided FMT catheter placement methods used in our center. To ensure the reliability and validity of the training, the session included hands-on practice with immediate feedback from experienced instructors, as well as a final assessment to confirm proficiency in both methods. Ultimately, all endoscopists became proficient in both methods and were able to choose either method for subsequent procedures. In this study, the included cases were divided into the DLC group (60 cases) and the CLB group (57 cases) based on the method selected by the endoscopists. Each patient underwent cecal intubation facilitated by colonoscopy, followed by fecal microbiota suspension injection. We collated and analyzed the basic clinical characteristics and operation-related outcomes from the medical records and corresponding endoscopist operational records of the patients. Due to the retrospective design, blinding of the endoscopist was unattainable; nevertheless, all outcomes were extracted and independently verified by two investigators (XL and QL collected clinical data. In case of any disputes regarding the results, the corresponding author will make the final decision).

The basic clinical characteristics of the two groups showed no significant differences (Table 1).

**TABLE 1:** Basic clinical characteristics of patients.

	DLC group, *n* = 60	CLB group, *n* = 57	*P*-value
Age (years), median (IQR)	33.0 [7.0–45.8]	16.0 [7.0–42.5]	0.389
Sex, *n* (%)			0.935
Male, *n* (%)	47 [78.3]	45 [78.9]	
Female, *n* (%)	13 [21.7]	12 [21.1]	
Body mass index (kg/m^2^), mean ± SD	19.5 ± 5.1	18.6 ± 4.2	0.299
Disease, *n* (%)			0.666
Autism	17 [28.3]	14 [24.6]	
Irritable bowel syndrome	28 [46.7]	32 [56.1]	
Crohn’s disease	1 [1.7]	3 [5.3]	
Ulcerative colitis	4 [6.7]	3 [5.3]	
Previous surgery^1^	3 [5.0]	1 [1.8]	
Others^2^	7 [11.7]	4 [7.0]	
Type of anesthesia, *n* (%)			0.715
Anesthesia	49 [81.7]	48 [84.2]	
Non-anesthesia	11 [18.3]	9 [15.8]	

^1^Previous surgery, including surgery-related radiation proctitis and enteritis.

^2^Others, including gastrointestinal indolent T-cell lymphoma, non-alcoholic fatty liver disease, rheumatoid arthritis, colorectal polyps, and eosinophilia.

### 3.2 Operation-related outcomes

#### 3.2.1 The effectiveness of the colonoscopically guided FMT catheter placement methods

Table 2 summarizes the catheter placement success rate and operation-related times of the colonoscopically guided FMT catheter placement methods. The catheter placement success rate for both the DLC and CLB groups was 100%. Regarding operation-related times, statistical analysis revealed no significant difference in cecal intubation time between the two groups (7.2 min vs. 6.8 min; *P* = 0.796). A statistical difference was observed in the first endoscopic clip securement completion time (3.7 min vs. 1.8 min; *P* = 0.006), indicating that the CLB group required less time for the first endoscopic clip securement compared to the DLC group. There were no significant statistical differences in withdrawal time (4.4 min vs. 4.3 min; *P* = 0.483) and total operation time (19.4 min vs. 16.0 min; *P* = 0.076) between the two groups.

**TABLE 2:** Catheter placement success rate and operation-related times of colonoscopically guided FMT catheter placement methods.

	DLC group, *n* = 60	CLB group, *n* = 57	*P*-value
Catheter placement success rate, *n* (%)	60 [100]	57 [100]	–
**Operation-related times**			
Cecal intubation time (minutes), median (IQR)	7.2 [4.5–10.9]	6.8 [5.0–11.2]	0.796
First endoscopic clip securement completion time (minutes), median (IQR)	3.7 [1.6–9.1]	1.8 [0.6–5.5]	0.006
Withdrawal time (minutes), median (IQR)	4.4 [3.1–8.1]	4.3 [2.9–6.0]	0.483
Total operation time (minutes), median (IQR)	19.4 [12.6–30.0]	16.0 [12.0–21.8]	0.076

#### 3.2.2 Endoscopist’s self-comparison

Additionally, we selected operational records from endoscopists who had practical experience with both types of FMT catheter securement methods and had performed at least five operations for each. A total of 5 endoscopists were included. We compared their operation-related times and found that for the first endoscopic clip securement completion time, there was a difference between the two securement methods for each endoscopist, with the CLB group taking less time than the DLC group (Supplementary Table 1).

#### 3.2.3 Paired analysis of two methods applied to the same patient

We performed a paired analysis of the operation-related times for patients who underwent both methods and found no statistical differences in cecal intubation time, withdrawal time, or total operation time between the two methods (Supplementary Table 2). A difference was observed only in the time for completion of the first endoscopic clip securement (*P* = 0.002).

#### 3.2.4 The safety of the colonoscopically guided FMT catheter placement methods

In the DLC group, 18.3% (11/60) of patients experienced mild adverse events, while in the CLB group, 17.5% (10/57) of patients had mild adverse events; no severe adverse events were reported in either group (Table 3). In the DLC group, 1.7% (1/60) of patients had catheter dislodgement. There was no significant statistical difference in the rate of dislodgement between the two groups.

**TABLE 3:** Adverse events following catheter placement.

	DLC group, *n* = 60	CLB group, *n* = 57	*P*-value
Adverse events	*n* = 11	*n* = 10	0.911
Catheter dislodgement, *n* (%)	1 [1.7]	0 [0.0]	1.000
Diarrhea, *n* (%)	4 [6.7]	4 [7.0]	1.000
Perianal pain, *n* (%)	1 [1.7]	2 [3.5]	0.612
Abdominal pain, *n* (%)	1 [1.7]	2 [3.5]	0.612
Fever, *n* (%)	2 [3.3]	0 [0.0]	0.496
Hematochezia, *n* (%)	1 [1.7]	1 [1.8]	1.000
Abdominal distension, *n* (%)	1 [1.7]	1 [1.8]	1.000

## 4 Discussion

### 4.1 Based on the background

Previous studies have confirmed that fecal microbiota transplantation (FMT) administered via colonoscopy is an effective method for FMT delivery in clinical practice (18). Compared with enema and nasogastric tube routes, colonoscopy-guided FMT administration has demonstrated high treatment success rates, low recurrence rates, and low adverse event rates. It allows for precise targeting of treatment, avoids the effects of gastric acid and digestive enzymes, and prevents respiratory aspiration (19). The capsule route for FMT has been reported to have treatment success rates and patient satisfaction comparable to colonoscopy (20). Although the capsule route is operationally simpler, colonoscopy remains advantageous in treating refractory cases such as recurrent Clostridium difficile infection and inflammatory bowel disease (21). The precise targeting capability of colonoscopy makes it more effective in some situations, especially in ensuring that the suspension directly acts on the lesion site (22).

The transendoscopic enteral tubing (TET) technique (23) reported by the team from Nanjing Medical University has provided valuable insights and serves as an important reference for our modified methods. Their approach has been shown to achieve high success rates and meet therapeutic goals. However, considering that the TET tubes used in clinical practice may be relatively expensive and that the specific operating steps might require a higher level of professional skill from endoscopists, we have explored some simplifications to their method. Our goal is to develop two approaches that retain effectiveness and safety while being more accessible to beginners and cost-effective. First, our method simplifies some of the operating procedures. In the TET technique, the colonoscope needs to be inserted through the anus twice: once to place the TET tube into the ileocecal region and again to secure the TET tube under colonoscopic guidance. Our method chooses to insert the colonoscope through the anus only once, securing the catheter as the colonoscope reaches the ileocecal region, which may reduce the potential adverse event rates associated with multiple intubations. Second, our method uses conventional gastric tubes as a catheter, which are inexpensive and easily accessible to most medical institutions and may be suitable for widespread clinical application.

### 4.2 Operation-related outcomes

#### 4.2.1 The effectiveness of the colonoscopically guided FMT catheter placement methods

The study results demonstrate that regardless of the method used to secure the catheter, the success rate of FMT catheter placement was 100%. This indicates that our methods are both capable of effectively securing the FMT catheter.

#### 4.2.2 Comparison of operation-related times

We conducted a detailed comparison of the operation times between the two methods. The two methods showed no significant differences in cecal intubation time, withdrawal time, and total operation time, demonstrating that both methods were effective in securing the catheter. However, we observed that the DLC method required statistically more time to secure the first endoscopic clip (3.7 min vs. 1.8 min; *P* = 0.006). Our analysis suggested that the increased time for the DLC method might be attributed to the stickiness of the loop and clip due to the presence of colonic fluid. Therefore, endoscopists must re-inspect and adjust the loop to ensure stable contact with the colonic wall (Supplementary Video 3), preventing displacement after securement due to patient movement or natural colonic motility. Despite this, the difference in this time had a minimal impact on the total operation time (19.4 min vs. 16.0 min; *P* = 0.076) and did not significantly affect the endoscopists’ ability to secure the FMT catheter. The shorter time required for the first endoscopic clip securement with the CLB method may be particularly advantageous in high-volume centers or for less experienced operators, which provides efficiency gains by ensuring the stability of the catheter and colonoscope in advance, potentially reducing the potential for errors during complex technical operations in colonoscopically guided FMT. Further validation through prospective studies is needed.

To more accurately assess the differences between the two methods, we employed two analyses. First, the self-comparison analysis of endoscopists allowed for a direct comparison of operation-related times between the two methods. This analysis aimed to reduce potential biases due to individual preferences or skill levels of the endoscopists, thereby compensating for the lack of randomization in our study design. Second, among the 117 cases included in the final analysis, some patients underwent multiple FMT treatments. To address the issue of non-independence of observations, we performed a paired analysis for patients who received both methods. This analysis aimed to minimize the influence of individual patient characteristics (such as baseline health status and physiological features) on the results, thereby providing a more accurate assessment of the differences between the two methods. The results showed that the two methods were comparable in terms of cecal intubation time, withdrawal time, and total operation time, further confirming their effectiveness in securing the catheter.

#### 4.2.3 The safety of the colonoscopically guided FMT catheter placement methods

No severe adverse events were reported in either group after the surgical procedures, and the minor discomfort experienced by the patients could be resolved spontaneously. There was only one case of catheter dislodgement in the DLC group, which occurred because a patient removed the catheter by himself. In our endoscopy center, the duration of FMT catheter placement for patients undergoing fecal microbiota transplantation does not exceed 3 days, and the catheter is actively removed after the completion of the fecal microbiota transplantation suspension injection for one course of treatment. Therefore, we have not focused on the retention time of the catheter and its potential impact on patient comfort/satisfaction for long-term treatment. In the future, we hope to pay more attention to improving patient-centered outcomes (such as long-term FMT treatment effects, patient comfort/satisfaction, etc.).

#### 4.2.4 Limitations and future directions

From a scientific perspective, the observed statistical results in this study may be subject to false negatives or false positives due to the small sample size and single-center design. Future multi-center studies could help to validate our results across different patient populations and clinical settings. By including multiple centers with diverse patient demographics and varying levels of endoscopist experience, we can better account for potential biases and ensure that our results are applicable to a broader range of clinical scenarios.

In summary, both methods can be adopted.

## 5 Conclusion

The two modified colonoscopically guided fecal microbiota transplantation (FMT) catheter placement methods provide some adoptable suggestions for the clinical promotion of TET technology. These methods are easy to master and cost-effective. Both methods can safely and effectively secure the FMT catheter, meeting the needs of patients who require multiple FMT treatments in a short period. The modified methods in this study need to be further verified through large sample randomized controlled trials.

## Data Availability

The raw data supporting the conclusions of this article will be made available by the authors, without undue reservation.
