# Supplementary material for: Two modified colonoscopically guided fecal microbiota transplantation catheter placement methods: a retrospective study (with video)
Source: Front Med (Lausanne). 2025 Oct 3;12:1641325. doi: 10.3389/fmed.2025.1641325 (PMC12531166; doi:10.3389/fmed.2025.1641325)
Supplement: Supplementary file 1 [file Table_1.docx]

Supplementary Material

# Supplementary Tables

**Supplementary Table 1. Comparison of Operation-related times for endoscopists.**

| Endoscopist | Group | Case，n | Cecal intubation time (minutes),  median (IQR)/mean ± SD | P value | First endoscopic clip securement completion time (minutes),  median (IQR)/mean ± SD | P value | Withdrawal time (minutes),  median (IQR)/mean ± SD | P value | Total operation time (minutes),  median (IQR)/mean ± SD | P value |
| --- | --- | --- | --- | --- | --- | --- | --- | --- | --- | --- |
| 1 | DTF | 8 | 10.0[6.1-11.1] | 0.583 | 4.4[1.8-10.8] | 0.007**^*^** | 9.3[8.1-21.4] | 0.015**^*^** | 30.2±14.8 | 0.035**^*^** |
|  | CCB | 15 | 7.1[5.7-13.4] |  | 0.8[0.4-1.3] |  | 5.4[4.3-8.9] |  | 16.4±6.4 |  |
| 2 | DTF | 9 | 5.8±2.7 | 0.042**^*^** | 5.1[2.9-12.2] | 0.033**^*^** | 6.5[4.1-8.5] | 0.019**^*^** | 16.9[14.6-19.4] | 0.894 |
|  | CCB | 5 | 13.2±5.8 |  | 2.8[0.1-3.6] |  | 1.9[0.8-4.6] |  | 17.1[13.5-22.4] |  |
| 3 | DTF | 8 | 7.5[6.0-11.2] | 0.965 | 8.3[3.0-22.6] | 0.003**^*^** | 3.1[1.9-12.0] | 0.533 | 28.5[13.2-42.3] | 0.091 |
|  | CCB | 10 | 8.0[5.8-15.8] |  | 0.7[0.5-2.7] |  | 3.8[2.1-6.6] |  | 12.5[10.9-22.6] |  |
| 4 | DTF | 13 | 5.2[3.7-11.5] | 0.167 | 6.1[3.8-17.2] | 0.027**^*^** | 3.7±1.1 | 0.397 | 21.9±10.8 | 0.980 |
|  | CCB | 5 | 9.1[5.8-25.0] |  | 1.4[0.7-4.8] |  | 5.4±4.1 |  | 22.0±12.0 |  |
| 5 | DTF | 9 | 6.3[4.4-12.2] | 0.814 | 2.5[2.4-11.6] | 0.033**^*^** | 3.6[2.8-9.6] | 0.556 | 21.4[14.3-25.6] | 0.077 |
|  | CCB | 6 | 7.3[2.5-12.6] |  | 1.3[0.7-2.5] |  | 3.5[2.3-6.1] |  | 11.0[7.9-19.2] |  |

*: Indicates a P < 0.05, suggesting a statistically significant difference

**Supplementary Table 2. Paired analysis of two methods applied to the same patient.**

| Patient | Cecal intubation time(minutes) | | | First endoscopic clip securement completion time (minutes) | | | Withdrawal time(minutes) | | | Total operation time (minutes) | | |
| --- | --- | --- | --- | --- | --- | --- | --- | --- | --- | --- | --- | --- |
|  | DLC | CLB | Difference | DLC | CLB | Difference | DLC | CLB | Difference | DLC | CLB | Difference |
| 1 | 10.0 | 7.1 | 2.9 | 5.6 | 0.6 | 5.0 | 8.1 | 8.9 | -0.8 | 23.7 | 16.6 | 7.1 |
| 2 | 10.6 | 19.5 | -8.9 | 3.2 | 0.8 | 2.4 | 10.1 | 11.2 | -1.1 | 23.9 | 31.5 | -7.6 |
| 3 | 8.0 | 13.4 | -5.4 | 5.1 | 0.3 | 4.8 | 5.8 | 5.4 | 0.3 | 18.9 | 19.1 | -0.3 |
| 4 | 10.4 | 7.5 | 2.9 | 4.1 | 1.3 | 2.8 | 4.5 | 4.0 | 0.6 | 19.0 | 12.8 | 6.2 |
| 5 | 6.8 | 10.9 | -4.2 | 1.6 | 0.5 | 1.2 | 8.5 | 4.6 | 3.9 | 16.9 | 16.0 | 0.9 |
| 6 | 7.6 | 6.2 | 1.4 | 1.3 | 1.8 | -0.5 | 2.6 | 10.6 | -7.9 | 11.5 | 18.6 | -7.1 |
| 7 | 9.1 | 5.7 | 3.5 | 2.5 | 0.4 | 2.1 | 3.1 | 5.3 | -2.2 | 14.7 | 11.3 | 3.4 |
| 8 | 3.7 | 4.1 | -0.4 | 4.7 | 1.2 | 3.6 | 2.3 | 4.0 | -1.7 | 10.8 | 9.3 | 1.5 |
| 9 | 6.3 | 6.5 | -0.3 | 2.1 | 1.0 | 1.1 | 11.5 | 3.5 | 8.0 | 19.8 | 11.0 | 8.8 |
| 10 | 7.9 | 5.8 | 2.2 | 2.3 | 0.8 | 1.5 | 11.2 | 8.0 | 3.2 | 21.4 | 14.5 | 6.9 |
| P value | - | - | 0.644 | - | - | 0.002^*^ | - | - | 0.874 | - | - | 0.302 |

*: Indicates a P < 0.05, suggesting a statistically significant difference.
